# A study to investigate the implementation process and fidelity of a hospital to community pharmacy transfer of care intervention

**DOI:** 10.1371/journal.pone.0260951

**Published:** 2021-12-28

**Authors:** Sarah M. Khayyat, Zachariah Nazar, Hamde Nazar

**Affiliations:** 1 School of Pharmacy, Newcastle University, Newcastle upon Tyne, United Kingdom; 2 Department of Clinical Pharmacy, Faculty of Pharmacy, Umm Al-Qura University, Makkah, Saudi Arabia; 3 Clinical Pharmacy and Practice Department, College of Pharmacy, QU Health, Qatar University, Doha, Qatar; National Taiwan University College of Medicine, TAIWAN

## Abstract

**Background:**

Hospital to community pharmacy transfer of care medicines-related interventions for inpatients discharged home aim to improve continuity of care and patient outcomes. One such intervention has been provided for seven years within a region in England. This study reports upon the implementation process and fidelity of this intervention.

**Methods:**

The process evaluation guidance issued by the Medical Research Council has informed this study. A logic model to describe the intervention and causal assumptions was developed from preliminary semi-structured interviews with project team members. Further semi-structured interviews were undertaken with intervention providers from hospital and community pharmacy, and with patient and public representatives. These aimed to investigate intervention implementation process and fidelity. The Consolidated Framework for Implementation Research and the Consolidated Framework for Intervention Fidelity informed interview topic guides and underpinned the thematic framework analysis using a combined inductive and deductive approach.

**Results:**

Themes provided information about intervention fidelity and implementation that were mapped across the sub processes of implementation: planning, execution, reflection and evaluation, and engagement. Interviewees described factors such as lack of training, awareness, clarity on the service specification, governance and monitoring and information and feedback which caused significant issues with the process of intervention implementation and suboptimal intervention fidelity.

**Conclusions:**

This provides in-depth insight into the implementation process and fidelity of a ToC intervention, and the extant barriers and facilitators. The findings offer learning to inform the design and implementation of similar interventions, contribute to the evidence base about barriers and facilitators to such interventions and provides in-depth description of the implementation and mechanisms of impact which have the potential to influence clinical and economic outcome evaluation.

## Introduction

A recent rapid review of hospital to community pharmacy transfer of care (ToC) interventions in England, was unable to report on statistically convincing clinical outcomes for patients receiving care from a community pharmacist post hospital discharge service. This is due to the low quality design approaches adopted, small sample sizes and singularity of the study [1]. The wider literature, shows that community pharmacists can successfully identify and rectify medicine-related issues post-discharge [2, 3]. A recent systematic review and meta-analysis evidences that when community pharmacists take an active role in a post-discharge intervention, there is an associated statistically significant 40% reduction in hospital 30-day readmission rates. However, authors report this cautiously given the heterogeneity in intervention design and evaluation, low intervention fidelity and generally high risk of bias associated with low quality study designs [4].

It is widely acknowledged that policy makers, practitioners and researchers need evidence from high quality evaluation to identify interventions which are effective, and also understand how to optimise those that are not. Process evaluations undertaken alongside rigorous experimental outcome evaluations can provide more detailed, valuable information to inform policy and practice [5]. Moore at al. have depicted that process evaluations examine aspects of implementation, mechanisms of impact and context. This allows more thorough appreciation of the cause and effect of an intervention, paying due consideration to the agency of implementers and participants and the intervention context, where is its implemented and experienced [6].

Given the mixed findings from ToC interventions, process evaluations are even more crucial to understand if failures or suboptimal outcomes are attributable to the intervention itself or to implementation practices; whether the intervention outcomes differ across the target population and investigate if effectiveness would be different if delivered in another context.

In this study, the focus is on a ToC intervention that was initiated in 2014 in the North East of England. Specific detail about the conception and design of the intervention is reported in a formative service evaluation following the first year of implementation [7]. This intervention with limited outcome evaluation, has contributed to the change in policy within England’s National Health Service (NHS) Community Pharmacy Contract, where a newly commissioned Discharge Medicines Service (DMS) was rolled out in early 2021 [8].

This study aims to investigate the implementation process and fidelity of this early ToC intervention in the North East. Findings are anticipated to elucidate how aspects of implementation influenced the intervention cause and effect, which could be valuable for policy-makers involved in the implementation, optimisation and evaluation of similar initiatives, such as the DMS.

## Methods

### Aim

To investigate the implementation process and fidelity of a ToC intervention.

### Design

The Medical Research Council (MRC) guidance [5] for process evaluations informed this study. The MRC network focus on the development of guidance for the conduct of population health research. The guidance for process evaluations reflects the recognition that for intervention evaluations of effectiveness to inform policy and practice, there is a need to understand how interventions are implemented, their causal mechanisms and how context impacts effectiveness [5].

#### Logic model development to define the intervention and underpinning assumptions

The first step in a process evaluation is to define the intervention and clarify the key assumptions. Logic models have been proposed as a diagrammatic representation of the intervention to display the clear and logical links between the intervention’s resources, activities and outcomes [8].

Previous evaluation of this ToC [7], included the explicit description of the intervention using the Template for Intervention Description and Replication (TiDIer) checklist [9]. However, given the lapse in time, this description was revised through replicating the previously reported method to capture any modifications. In brief, semi-structured interviews using a topic guide informed by the TiDIER checklist was undertaken with key informants (n = 3) who could provide detail on the design, implementation, operation, modifications since conception and monitoring of the intervention (The topic guide is included in the S1 Guide). These participants were identified as members of the original project team who designed and implemented the ToC. These representatives were recruited for an interview via email invitation, accompanied with a participant information sheet and consent form, through the ToC project group.

An initial set of intervention assumptions, activities and intended outcomes were derived from the triangulation of the data from reviewing the previous study reporting on this intervention and analysing the interview transcripts and the completed TIDier checklist (included in S1 Checklist).

Through organisation of this data, a preliminary logic model was developed.

Following the investigation of implementation (as described below), the logic model was refined to highlight where assumptions had been corroborated or otherwise, and therefore provide a more accurate representation of the intervention.

#### Investigating the intervention implementation process and fidelity

The MRC guidance set out the key components of process evaluation (context, implementation, mechanisms of impact) and the relationships between them and between the description of the intervention and the outcome. There is an increasing number of implementation science studies in the field of pharmacy to better understand how and why interventions do or do not work, and therefore add more richly to the evidence base. Researchers have employed a range of theories and frameworks to quantify and better understand context, implementation and mechanisms of impact. In this study the focus is on implementation; specifically how delivery is achieved and what is delivered in terms of fidelity.

*Conceptual frameworks*. The Consolidated Framework for Implementation Research (CFIR) [10] and the adapted Consolidated Framework for Intervention Fidelity (CFIF) [11] has underpinned this study’s data collection and analysis. The CFIR is a valid, useful practical guide for investigating different contexts and exploring potential barriers and facilitators affecting how delivery is achieved. It covers five main domains that have been associated with practical implementation (Table 1).

**Table 1:** The CFIR domains.

CFIR Domains	Exemplar items
Intervention characteristics	The advantage of implementing the ToC intervention, its adaptability and its complexity
Outer setting	The availability of external strategies and policies to spread the use of the ToC intervention
Inner setting	The nature and quality of networks and communications within the setting affecting the implementation, and the availability of resources dedicated to implementation
Characteristics of the involved individuals	The individual knowledge and beliefs about the ToC intervention
Process of implementation	The education and training for implementing the ToC intervention and evaluating the progress and quality of the implementation

The domains of the framework take a holistic, systems-thinking approach to understand the implementation of an intervention [12–14].

**Fig 1:**
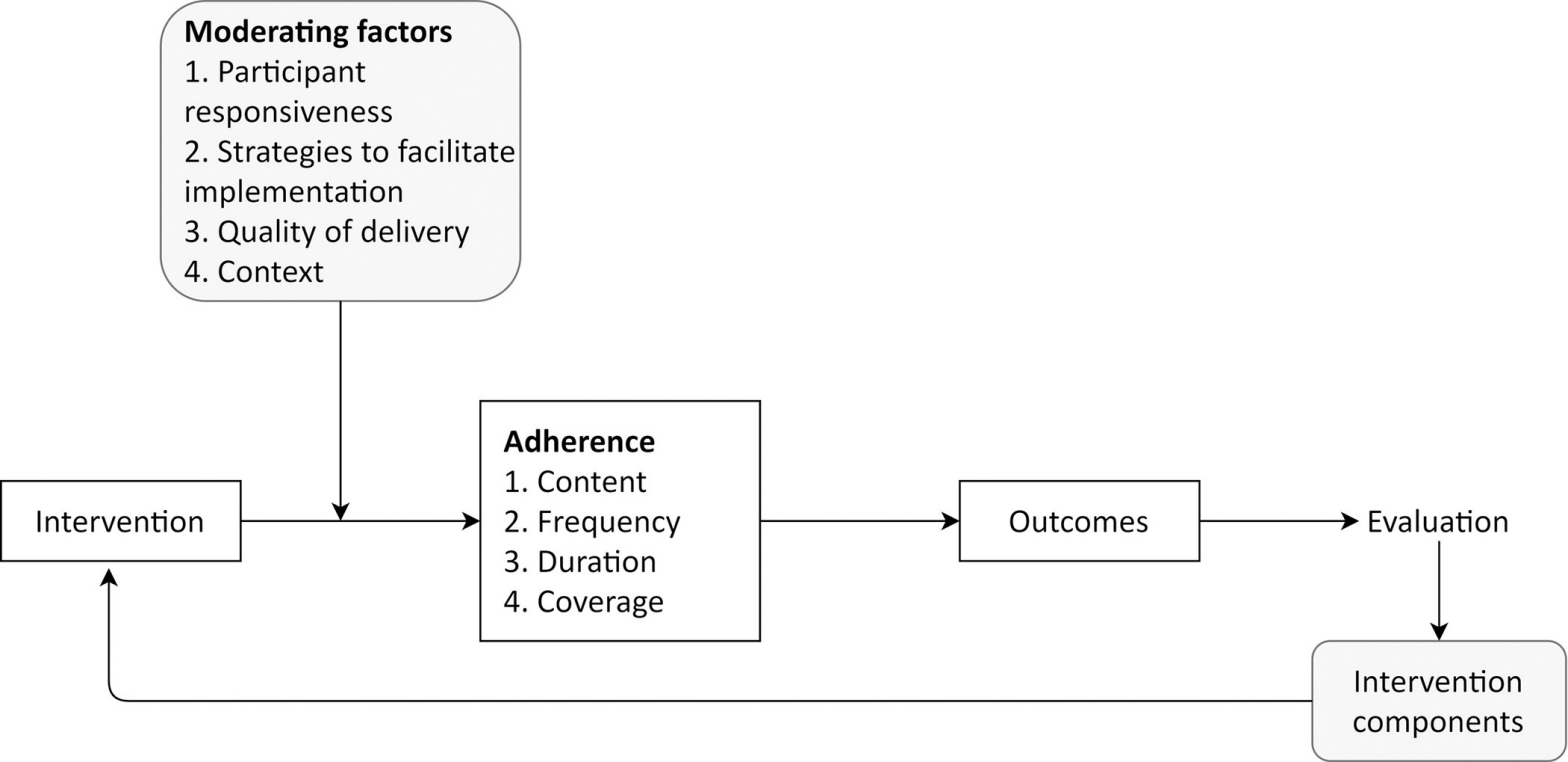
The adapted consolidated framework for intervention fidelity for the ToC intervention.

In order for an effective intervention to achieve successful outcomes, implementation fidelity (what is delivered) needs to be high. The adapted Consolidated Framework of Intervention Fidelity (CFIF) (Fig 1) offers a conceptual framework that enables another multi-faceted evaluation of implementation fidelity considering both the intervention and its delivery [11]. The CFIF has significant overlap with the CFIR but the former enables a more granular scrutiny of the how intervention integrity contributes to outcomes. As such, a greater insight can be gained about how elements of context and intervention causally link to achieving and recording intervention outcomes.

Adherence to intervention protocol encompasses the content and dose of the intervention, i.e. all active components, being received by all potential end users as often and for as long as it should have been. However, adherence, i.e. high implementation fidelity, is influenced and can be moderated by factors affecting the delivery process, such as facilitation strategies, quality of delivery, context and participant responsiveness [11].

*Stakeholder interviews*. Stakeholder, or key informant interviews were the selected methodology to investigate implementation process and fidelity. Key informants were those identified with the relevant experience of ToC to provide detailed information about its implementation, operation and ongoing delivery. These included project team members (PTM; the designers and managers), hospital pharmacy staff (HPS; the referrers), community pharmacists (CPs; the receivers of the referral and providers of post-discharge care) and patients and the public (the end users). Table 2 outlines the recruitment stratgies of these participants.

**Table 2:** The sampling and recruitment strategies for stakeholders interviews.

Stakeholder	Sampling strategy	Recruitment strategy
**Project team members**	Purposive sampling from the North East ToC project team membership	Participants were known the research team and invited via email including a participant information sheet and consent form to be completed and returned to indicate participation.
**Hospital staff**	Convenient sampling from the two hospital sites providing the ToC service in the North East	Participants invited by email including a participant information sheet and consent form via a gatekeeper. Willing participants returned the completed consent form to the researcher to indicate participation.
**Community pharmacists**	Convenient sampling from the 498 community pharmacies providing the ToC service in the North East	Participants were sent the invite, participant information sheet and consent form via a centralised messaging service. Willing participants returned the completed consent form to the researcher to indicate participation.
**Patients and the public**	Convenient sampling from advertising on two organisational websites (Diabetes UK and Voice®) (more information published elsewhere) [15]	An advert was posted on the appropriate Diabetes UK website as an invite to the study by contacting the research team directly.
		The invite for the research, including participant information sheet and consent form were emailed to the membership of Voice® as part of a regular email communication. Willing participants returned the completed consent form to the researcher to indicate participation.

The topic guides for the PTM, HPS and CPs (included in S1–S3 Guides) were all informed by the key informant guide by O’Haire et al. [16], the CFIR [10] and the preliminary logic model.

The topic guide for the PP was informed by a rapid review of the literature (included in S4 Guide). The full PP qualitative investigation and accompanying COREQ [15] is published elsewhere [17].

Interviews were conducted either face-to-face or over the phone, audio-recorded with consent and then transcribed verbatim by one researcher [SMK]. Transcripts of the interviews were subject to thematic framework analysis providing opportunity for exploratory and explanatory interpretations. A combination of inductive and deductive coding was used [18]. For the interviews with PTM, HPS and CPs, *a priori* codes were derived from the CFIR [10] and the CFIF [11] and for the PP interviews *a priori* codes were informed from a preceding rapid review of the literature as reported elsewhere [17].

Data management and coding was conducted in NVivo. One researcher [SMK] transcribed the interviews as part of the familiarisation with the participant accounts, another two researchers [HN, ZN] read all accounts and the team met to discuss and agree on perceptions of the stakeholder experiences. In addition, 10% of the transcripts were independently coded in duplicate [HN, ZN], and discussions were used to again agree on coding framework and resolve any inconsistencies. Validity of findings was enhanced through the use of constant comparison undertaken throughout data collection and analysis where data was compared and contrasted from interviews within the same stakeholder group and from interviews between different groups. Contradictory evidence, or deviant cases were sought out, examined and accounted for in the analysis [19].

Participant recruitment continued until saturation was achieved. The four models of saturation as described by Saunders et al. [20], were considered at different stages of the research process as shown in Table 3.

**Table 3:** Models of saturation considered in this study.

Data saturation	Occurred at the data collection phase where new data tended to be redundant of data already collected. There were no new themes generated after the 8^th^ interview with HPS, 7^th^ interview with CPS, and after the 9^th^ interview with PP; so it was deemed that data collection had reached a saturation point. Two more interviews were conducted with each group of participants to check and confirm that no new themes were emerging.
Theoretical saturation	Occurred when the complete range of constructs that explored the ToC intervention was fully discussed with the participants and represented by the data, driven by the notion of theoretical sampling. Therefore, the determinant of adequate sampling related to the degree of development or completeness of theoretical categories in the process of analysis.
*A priori* thematic saturation	Was considered when the pre-determined codes or themes from the CFIR and CFIF were adequately represented in the data.
Inductive thematic saturation	Was considered when there were no new themes or codes emerging in the process of analysis.

The COREQ checklist [15] has been included in S2 Checklist to capture relevant detail about the conduct of this qualitative work. The strategies to uphold the four principles of establishing trustworthiness in qualitative research as articulated by Lincoln and Guba [21], were embedded in the conduct of the research and are articulated in the COREQ checklist [15].

Ethical approval for interviewing each of the stakeholder groups was sought individually and was successfully awarded Institutional ethical approval from the Newcastle University, Faculty of Medical Sciences Ethical Committee (PTM, HPS and CPs interviews REF 1497/4636, PP interviews REF 13444/2018), and Research and Design approval from the hospital. The ethical review considered the work not falling in the remit of requiring NHS Health Research Authority approval, especially as the patients and public were not recruited via the NHS.

## Results

**Fig 2:**
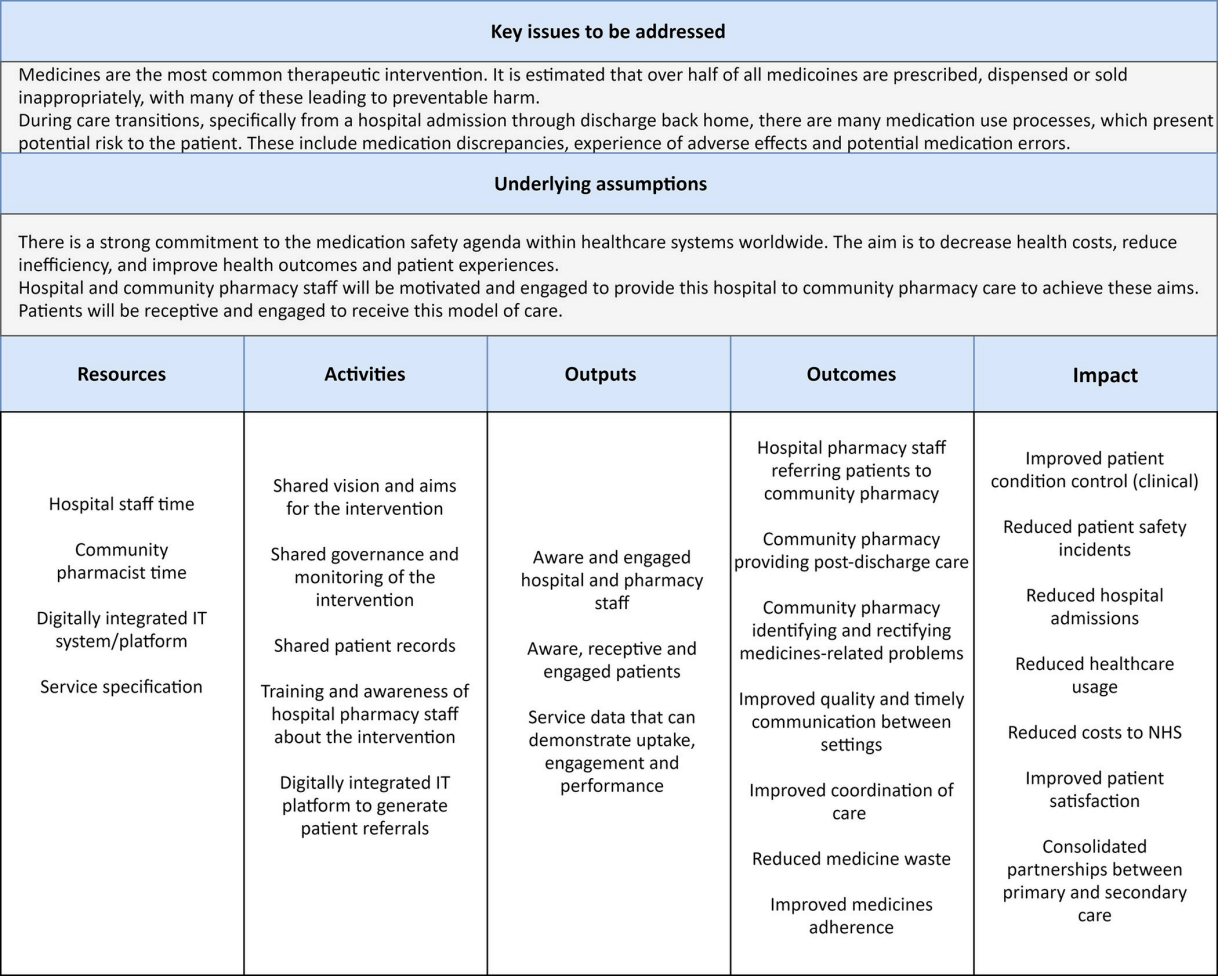
A logic model of the ToC service.

The data from previous research, completed TiDiER checklists and interview transcripts allowed the development of the ToC logic model (Fig 2).

Over the period from 20/07/2018 to 19/08/2019 interviews with three PTM, ten HPS, nine CPs and eleven PP were undertaken either face-to-face or on the phone. Interviews lasted for 47 mins ±14 mins. The demographic details of these participants is included in S1 Table.

PTMs were consulted two further times after their initial interview to clarify, check and obtain further detail about aspects of ToC that were discussed with HPS and CPs.

There were two key constructs of the CFIR that were of significant interest as they resonated and connected all interviewee responses. Firstly, there was agreement amongst the interviewees that the ToC intervention was *compatible* (CFIR: inner setting) [10] in terms of values of meaning with the expectations of pharmacists working in primary and secondary care within the healthcare system. The ToC was considered, in principle, to fit within existing workflows and systems.

“*I think probably historically communication has not been that great between primary and secondary care and it’s something that has always some kind of stress*. *It’s always been an issue*. *We need to improve the flow of information between the two to improve joining up the care for safety of the patients*. *I think it’s* [the ToC intervention] *sort of helping with that to helping joining up the care and improving communication*. *It does make sense to do it”*, *04HPS*

“*I think it is an excellent service*. *Because I remember in the past when my mother had a stroke and she was in the hospital for quite a long time*, *when she came out*, *there was no form of contact*, *even with the doctor*. *The aftercare*, *there was nothing*. *(*..*)* [With the ToC intervention] *They did call me*, *but that was for maybe a 10-minute talk*, *which is very kind of them to do this*. *I did say thank you very much for this because you have made me feel calmer and more confident*. *It felt like a natural flow of care”*, *02PP*, *with LTC*

However, the second common construct: *knowledge and beliefs of the intervention* (CFIR: characteristics of individuals) [10], highlighted paradoxically that understanding and perceptions of the specific working and value of ToC was varied and, in some cases, conflicting across the stakeholder groups. For example:

“*I think the service is a really important help*. *If we get that transfer of care right*, *the patients are looked after*, *not just from when they come in the door but when they go out of the door here in hospital*, *or when they go home*, *someone is looking after them*. *We are doing the same job but just in different sectors*. *We have to work together*. *So I think it is really valuable”*, *07HPS*

“*It just gives us more information about what the patient’s journey has been*. *So*, *it lets us know that they’ve been in hospital*. *They’ve been discharged with a new medication*, *that sort of thing*, *which we didn’t always necessarily know in the past with general patients*. *Medibox patients*, *yes*, *we knew they were in hospital and when they were discharged*, *but normally with*, *other patients*, *we don’t know”*, *03CP*

“*I actually think if we had better discharge letters*, *you probably would not need PharmOutcomes®*. *(*..*) I do not really understand the point in referring because at the end of the day community pharmacists can’t actually do anything”*, *06HPS*

**Fig 3:**
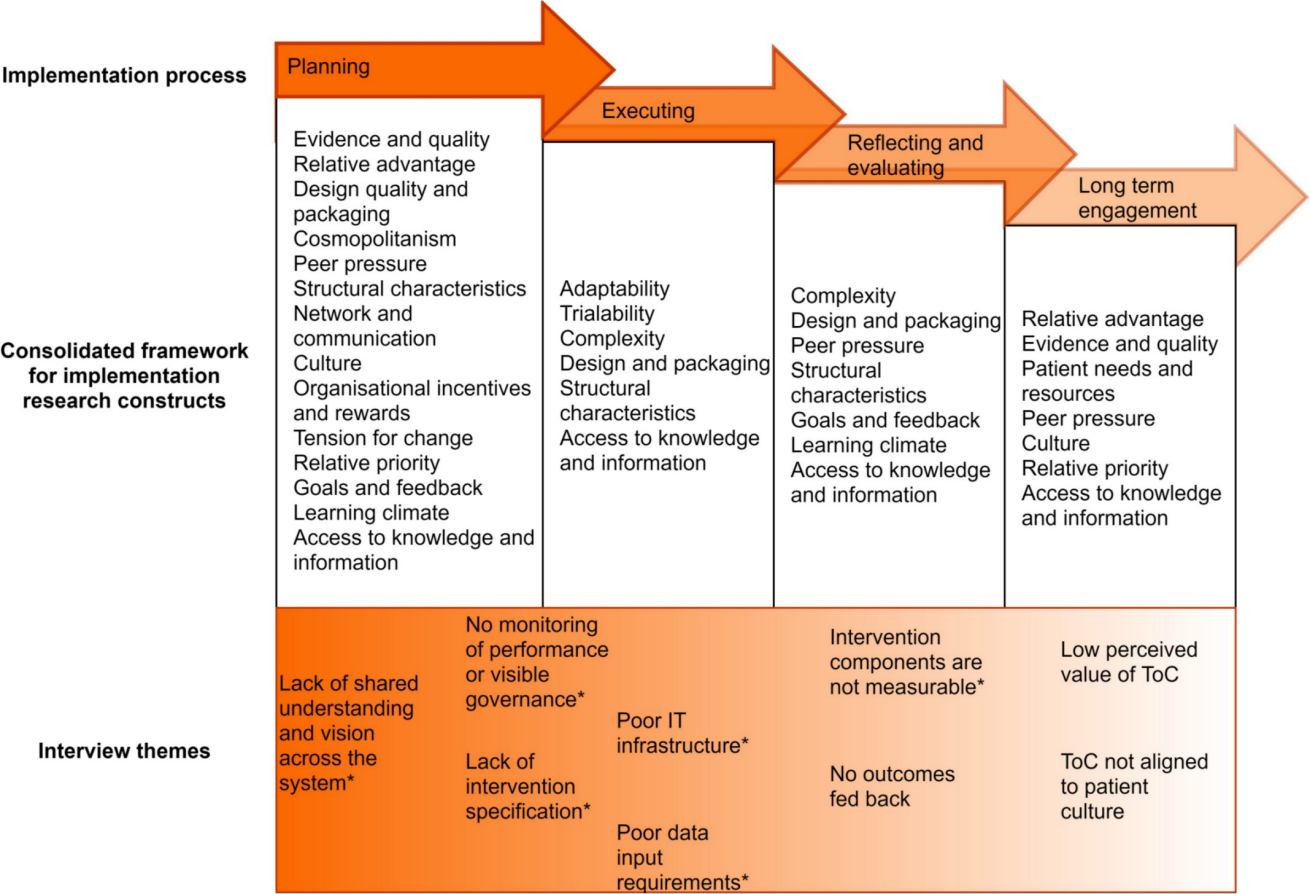
The main themes from stakeholder interviews, how they map to the constructs of the CFIR and how they relate to the implementation framework. Themes asterixed (*) are also those which provide insight on implementation fidelity.

The remaining main themes are presented in Fig 3 and are discussed below. These have been framed by the CFIR fifth domain: *implementation process* [10]. The CFIR presents the implementation process as an interrelated series of sub processes, i.e. planning, engaging, executing, reflecting and evaluating, that do not necessarily occur sequentially as they often happen simultaneously at different levels of the system [10]. The figure (Fig 3) aims to illustrate the overlap and interrelationship between these sub processes. Notably, ‘engaging’ is an underlying factor but also its longer-term significance depends upon the credibility of all other sub processes (planning, executing, reflection and evaluating).

### Planning

Seven years after conception and intervention delivery, there was a lack of coherent vision about the value of ToC between the interviewees. The PTM avidly articulated the need, significance and potential impact of ToC.

“*There are plenty of wards that don’t have pharmacist cover*. *So in those places there could be plenty of people slipping through the gaps… People who could really benefit from a service like this”*, 07HPs

HPS were however, ambivalent due to lack of training, awareness, feedback of ToC and appreciation of the capabilities of their CP counterparts.

“*I don’t know what … one community pharmacy can offer might not be what another pharmacy can offer*. *So you don’t want to ask them to do something they can’t do*. *(…)”*07HPS

“*I find that maybe there are some patients I think who would benefit from some care after a hospital stay*, *but I am just unsure a community pharmacy can really do*!*”* 08HPS

CPs concurred that ToC was necessary and welcomed given they were often ignorant to their patients’ hospital admissions and discharge due to exclusion from primary-secondary (and vice versa) care communication.

“*Sometimes*, *well usually*, *the first time we hear that a patient has been in hospital is when we give them their next set of meds*, *which is usually a few weeks after their discharge*.*”* 03CP

Patients were positive and open to the involvement of CPs in their care post-discharge, however, raised concerns about the perceived limited capabilities of CPs and their restricted access to patient’s information. *“I mean I like my pharmacist*, *so talking to him after being in hospital would be fine*. *But will he have all my information*? *Like all the stuff that went on in hospital so he can get an idea where I’m at*?*”* 04PP

Also, more widely, ToC was still only being initiated in hospital by pharmacy staff. No other healthcare professional had been engaged with to widen service provision, improve patient recruitment and normalise practice. Patient referral into the ToC intervention was only possible if the pharmacy team were directly involved in their care, which was not standard practice on all hospital wards.

“*No one other than the pharmacy knows about it*. *So there should be other places where it could be used by nursing staff or doctors or medical staff*. *(*..*) there are definitely certain areas [wards] that could benefit from it…”*07HPS

“*… quite a few wards uncovered by the pharmacy*. *They are covered remotely for discharges*, *but there is not a pharmacist or technician who visits the ward and sees the patients”*, 03HPS

Notably, despite the project team having membership from across the region, the ToC had not been adopted by other hospitals. The PTMs opined that the lack of outcome data limited influencing wider change in the system, e.g. gaining buy-in from other hospital staff to generate ToC referrals, and encouraging intervention adoption at other hospitals in the region.

“*There was that one evaluation but other than that there has not been a good level of regular evaluation that we can show other hospitals in the area*.*”* 07HPS

The HPS and CPs reported that there was no service specification available that would articulate the clear aims and objectives of ToC, formalise collaborative working and transparently describe the requirements of intervention delivery. As such there was practice variation within the hospital between HPS, and between CPs.

“*Because we do not have guideline and policies on when to do a referral and when not to do a referral”*, 05HPS

“*Different teams do different things*. *So even two people in the same team will do PharmOutcomes differently because we do not have any standards that say what’s expected”*, 05CPS

Patients related their personal confusion, where they had previously experienced inconsistent types of services and care from different pharmacies.

“*It’s quite common really*, *like some pharmacies do these services as standard*, *and then you walk into another pharmacy and you can just about get some shampoo*!*”* 03PP

Initially, the performance of the ToC intervention was monitored by both the hospital and community pharmacy project team leads. This governance was a strategy to facilitate implementation and did provide hospital staff with knowledge, feedback and motivation to engage and value the intervention. This governance and monitoring however ceased after the first two years of delivery. The absence of; incentivisation; goals and feedback; visibility and tangible information about impact on patients led to HPS ambivalence and dismissal of ToC.

“*That’s a bit of a gap in our practice (…)*, *maybe we do not use it as well as we should because we have not had a refresher to say these are the people you should be targeting”*, 07HPS

*“We do not have a lead on [the service]*. *We need someone to lead on it*, *write the procedure*, *to say how we need to use it and then to disseminate all this to everyone*.*”* 05HPS

*“I think it does need evaluations and re-working*. *I have the feeling that we created the service*, *and …this great work… but then it kind of feels like it’s just stopped*. *We have not pushed it further to make it better by adding those extra bits of information or making it less cranky*, *it’s just stopped”*, 07HPS

CPs reported having yet to receive significant numbers of referrals to their pharmacies for ToC to be a normalised daily activity. Also, the perceived deficiency in discharge information sent to CPs, had left CPs recording their post-discharge care in a restrained manner that did not clearly link to outcomes or impact on the patient.

*“So I think I have managed just one referral*, *like in the past 2 years*. *I mean that’s not good is it*?*”* 08CPS

The lack of evidence about CPs input and effect on patient care and outcomes contributed to the relatively low commitment of HPS to the ToC intervention.

### Executing

The integrated IT platform was a key facilitator for the generating and transmitting of a referral from hospital to community pharmacy. The auto-population of the fields from the existing hospital system helped reduce time required for and accuracy of data input. However, not all details about the patient’s hospital admission was automatically included, e.g. reason for admission, changed medication.

*“When they introduced the autopopulation of some of the fields*, *that was helpful and saved a lot of time*.*”* 05HPS

CPs expressed the requirement for a fuller discharge summary to facilitate a meaningful and valuable post-discharge intervention. The fields for data input from CPs were not mandatory and required dichotomous indication (yes/no) on processual outcomes such as receipt of referral, action of referral, advice on medicines provided. There was no requirement to record issues identified and/or addressed, readings from any clinical examinations, e.g. blood pressure, or feedback from patients. This elective data entry meant there was a lack of consistent data around intervention content, frequency and duration.

*“It felt a bit like a ‘tick box’ exercise*. *And given we did not have that much information*, *the data recording was quite sparse I think*.*”* 09CP

*“We are not sharing everything we know… and that’s not helpful*. *We should be sharing everything we can to try to make the transfer of care smoother”*, 07HPS

*“It sometimes*, *I think*, *depends on who writes it because sometimes you’ve got lots of details and that’s very helpful*. *Sometimes it’s quite short”*, 05CP

Also, there was no closed loop on the referral, where the hospital pharmacy staff would have the opportunity to review the outcomes of a referral they had generated.

*“I do think a barrier within the staff in hospital is that we send this form and we never see it again and you don’t know if they even had a conversation with the patient*, *let alone*, *if there was actually any positive feedback from it”*, 08HPS

The lack of information in both settings contributed to the vacillating engagement with the recruitment of patients into ToC and the level of activity and recording of that activity in community pharmacy.

*“Like we don’t get to hear any of the hospital side and the hospital doesn’t get to hear any of the community side*. *(*..*) The hospital and the community sides are two worlds apart*. *And they’re trying to connect them but we don’t know what they can see*, *and what they think we do and vice-versa”*, 02CP

### Reflection and evaluation

The data routinely recorded for the intervention did not support outcome (clinical or economic) evaluation or capture the impact on patients. There were no data fields to enable assessment of the four tenets of adherence: content, frequency, duration, and coverage.

*“I think the biggest barrier to us being fully on board…is the fact that it’s very one-sided*. *We never get any feedback*, *to how we are doing*, *whether what we are doing is good or bad*, *or leave any outcomes from the referrals that we make”*, 05HPS

*“Some people found it demoralising because you could send 4 or 5 and then they would always be rejected*, *and that’s quite demoralising if you did a lot of work*. *(*..*) People don’t like doing this if they don’t see any benefit”*, 07HPS

The project team also ceased to provide oversight, monitor and govern performance through the review and scrutiny of the service data.

### Longer term engagement

Longer term engagement was mostly dependent upon the initiators of ToC generating referrals, (HPS) and the receivers of ToC (PP), given that the providers (CPs) perceived the intervention as aligning with their standard role and responsibilities and fitting appropriately into existing workflows and systems. The PP and HPS, despite being positive about the involvement of CPs in patient post-discharge care, expressed low perceived value of ToC.

*“If we felt that it would stop people coming back to hospital*, *or that it would reduce our workload*, *if we could see that if you referred someone*, *it would reduce your workload by this amount*, *then we will be thinking ‘Oh*! *It actually works*. *That’s really worthwhile*. *Or if it reduces drug spending or any of those kind of things”*, 07HPS

HPS perceptions were justified by aforementioned reported issues relating to lack of training, awareness, clarity on the service specification, governance and monitoring and information and feedback from CPs.

*“So I think the feedback from the community and the hospitals should be put back in a pool so everybody can read it*. *It would give them an insight as to say*, *‘Well*, *actually we should start using this more’”*, 02CP

“Getting some positive feedback every now and again does make it feel like it’s worthwhile…having a lead on it and regular check ins or refreshers about it would be good drivers”, 08HPS

PP related that CPs were not widely regarded as integral healthcare practitioners. Their role in the supply and selling of medicines was accepted and acknowledged, however, PP claimed that for health-related issues, or where there is a deterioration in health, general practitioners were their favoured point of access.

### Refined logic model

**Fig 4:**
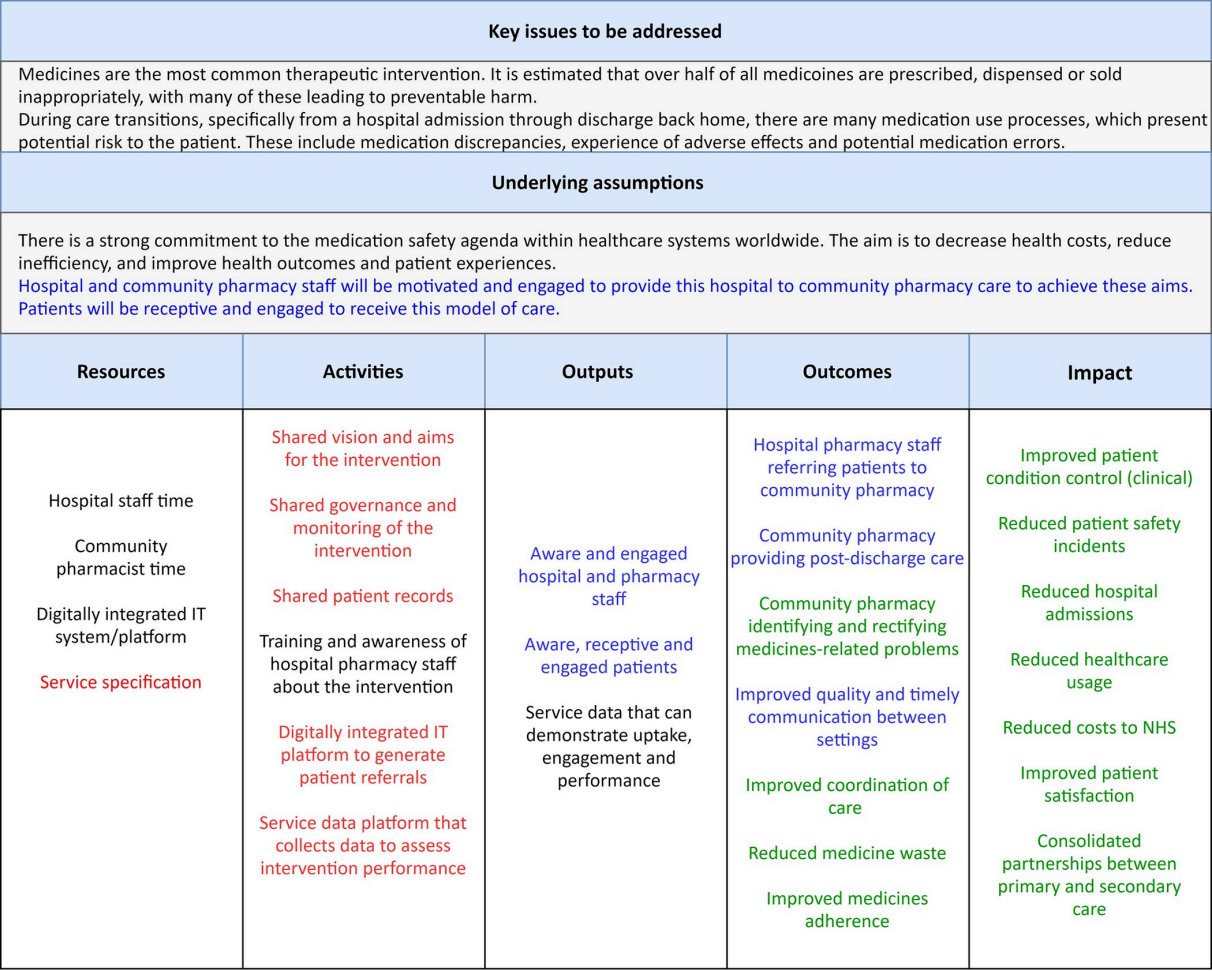
Refined logic model for the ToC intervention where assumptions have been confirmed (black), partially verified (blue), negated (red) or not captured in this study (green).

The initial logic model was reviewed in light of these findings. Fig 4 illustrates where assumptions have been verified, partially verified, negated, or have not been possible to capture by this study.

## Discussion

This study has presented in-depth contextual insight of a ToC intervention. The logic model framework has explicated initial assumptions that were tested through the data collection and analysis. The use of the CFIR [10] and CFIF [11] enabled systematic interrogation of the barriers and facilitators across the system, and particular scrutiny on the intervention implementation process and fidelity. The findings illustrate components of the intervention and the wider system that require attention to improve implementation within this context but also enhance the potential for diffusion and wider adoption. Namely, a clear service specification that articulates the aim and vision of the intervention but also facilitates the standardisation (improved fidelity) of the quality and content of intervention delivery and operation. This should be supported by ongoing training and awareness of the intervention until it is truly embedded and normalised into practice. The routine intervention data requirements needs to be appropriate to facilitate performance and outcomes assessment. This will provide evidence to drive intervention provision, wider diffusion and adoption, thereby impacting patient engagement and acceptability. Lastly, shared governance, patient records and monitoring of the intervention’s performance would also enhance wider buy-in, awareness, and engagement within the system. This will optimise the context and participant responsiveness to the intervention, leading to longer term sustainability.

A recent realist review presents a programme theory of what works, for whom and under which circumstances when pharmacists undertake medication reviews in primary care post-discharge [22]. The realist synthesis has a greater focus on the achievement of outcomes for patients rather than interrogating the implementation process. However, findings of our study concur with this programme theory in the following areas:

- Patients’ acceptance of community pharmacy post-discharge care will be improved where already trusted hospital staff make referrals and recommendations to CPs;
- Patients need to have prior positive experiences with CPs to improve perceived value and engagement with ToC interventions;
- Raising awareness of the value of CPs amongst healthcare practitioners in the wider system enhances intervention provision and engagement with referral generation; and
- Sufficiently detailed information sharing between primary and secondary care will facilitate CPs engagement in providing post-discharge care.

Our study provides further details about context, intervention and mechanisms that can be incorporated into this programme theory. These include: the need for a two-way sharing of patient data so that HPS can see the outcomes of their referrals, which will drive engagement with hospital referral generation, and the need for mechanisms and mandatory high quality data entry to enable regular service performance monitoring and review. This refined programme theory can be iteratively tested following a realist approach across multiple case studies as described by Fletcher et al. [23] In England, a recent rapid review found ten ToC interventions that could be considered as further case study sites for further development of the programme theory [1]. Findings from this process will add richness and granularity to the theory but also highlight aspects of the context, intervention and mechanism that are adaptable or inflexible. This will mean the subsequent evidence-based recommendations that can be made to intervention designers, commissioners and implementers are more specific to achieve successful implementation and positive patient outcomes.

This learning bears significant relevance in England at this time, where a new national DMS has been commissioned. The design, implementation and delivery of this new service can capitalise on these findings to improve the potential for success and sustainability.

This study is limited in that it is single-site in nature, which raises questions about the applicability outside this context. However, internationally, ToC interventions are of interest [24], so study findings are potentially of interest outside the healthcare system of England. The ‘thick description’ [21] provided will facilitate the assessment of transferability to other contexts, times and people.

## Conclusion

This study has highlighted deficiencies in a ToC intervention implementation process and fidelity. The granularity of this information is useful to optimise the onward delivery of the intervention and contributes to existing data and theory to inform the design, delivery and evaluation of similar interventions. The explanation of implementation also provides contextual information to better understand any outcomes measured via an evaluation. Future work should similarly follow guidance in evaluating complex interventions by undertaking a process evaluation alongside any outcome evaluation to understand the effectiveness of an intervention in the context it is being delivered and via the implementation processes by which it is introduced and delivered.

## Supporting information

S1 Checklist Intervention elements according to the TIDieR checklist. (PDF)

S2 Checklist Consolidated criteria for reporting qualitative studies (COREQ). (PDF)

S1 File Coding frameworks. (DOCX)

S1 Guide The interview guide with the project team members. (PDF)

S2 Guide The interview guide with hospital pharmacy staff. (PDF)

S3 Guide The interview guide with community pharmacists. (PDF)

S4 Guide The interview guide with patient and the public. (PDF)

S1 Table Demographic tables for PTM, HPS and CPs (a) and for PPs (b).
(PDF)
