# Correction: A study to investigate the implementation process and fidelity of a hospital to community pharmacy transfer of care intervention

**DOI:** 10.1371/journal.pone.0306784

**Published:** 2024-07-03

**Authors:** The PLOS ONE Staff

The second author, Zachariah Nazar, was incorrectly listed as the first author of this work. Sarah M. Khayyat is the correct first author. Sarah M. Khayyat and Zachariah Nazar should be noted as joint senior authors, contributing equally to this work.

The publisher apologizes for the error.

## References

[1] Khayyat SM, Nazar Z, Nazar H (2021) A study to investigate the implementation process and fidelity of a hospital to community pharmacy transfer of care intervention. PLoS ONE 16(12): e0260951. doi: 10.1371/journal.pone.0260951 34962937 PMC8714098

