# Supplementary material for: A study to investigate the implementation process and fidelity of a hospital to community pharmacy transfer of care intervention
Source: PLoS One. 2021 Dec 28;16(12):e0260951. doi: 10.1371/journal.pone.0260951 (PMC8714098; doi:10.1371/journal.pone.0260951)
Supplement: S1 Checklist — (PDF) [file pone.0260951.s001.pdf]

Additional file B. Intervention elements according to the TIDieR Checklist.

Item	Description
**1. Name of intervention**	Electronic transfer of care service (ToC) or electronic referral service or e-referral service or clinical handover.
**2. Rationale for intervention**	There was a need to develop a service that improved medicine management on discharge when the patients were returning home and re-starting their medicines because it was found that between 30-70% of patients had an error or an unintentional change to their medicines during their transfer of care. Besides, between 30-50% of patients had not taken their medicines as intended, which had affected their quality of life and might have resulted in disease complications or hospital admission. It has been shown that involving community pharmacists in the patients' care and having effective communication between healthcare professionals and with the patients themselves have avoided unnecessary hospital re-admissions, medicine-related problems and patient safety issues.
**3. Resources and materials**	- PharmOutcomes® was required as the referral software to make referrals from hospital to CP, and there was no associated cost, as CPs were already using it for other clinical service delivery and data capture.<br>- Both HPS and community pharmacists received training on the PharmOutcomes® platform about how to use the service. They also were distributed materials/screenshots to become familiarised with the PharmOutcomes® interface.<br>- HPS and community pharmacists received no extra payment or reimbursement for providing the service.<br>- There were no leaflets or advertising for the service. Patients were only asked about their agreement to be referred to their nominated CP for post-discharge care.<br>- The service leaders at the hospital encouraged their pharmacy staff to provide the service. The CP area managers were contacted by the chair of the Local Pharmaceutical Committee to inform them of the service and ensure they encouraged their community pharmacists to provide the service.
**4. Procedures**	**Hospital procedure:** HPS identified patients who would benefit from a post-discharge CP follow-up. The staff were using their clinical judgement to refer patients as there were no specific patient eligibility criteria. Then, patients were approached on the wards to get their agreement to be referred. HPS were entering the details of the consenting patient onto the PharmOutcomes® referral template.<br><br>**Community pharmacist procedure:** Community pharmacists accessed their PharmOutcomes® records regularly to check for referral requests sent from the hospital and to act upon them. They had the choice of either 'accepting' or 'rejecting' the referral. When they rejected the referral, they were prompted to provide a reason for this rejection. Alternatively, they could accept the referral

Item	Description
	and arrange for a follow-up consultation with the patient. Once community pharmacists had completed the follow-up, they were required (non-mandatory) to record their actions/activities in PharmOutcomes® under the section 'Complete the referral'.
**5. Providers**	In the hospital, both pharmacists and pharmacy technicians were required to refer patients as part of their usual role in the care of hospitalised patients. On receiving the referral request, community pharmacists were involved in following up the patients to provide the requested or needed post-discharge service.
**6. Mode of delivery**	HPS approached patients admitted onto wards for their agreement to be referred to their nominated CP. Then, the community pharmacists contacted the referred patients on the contact number provided by the HPS. Face-to-face and phone-call consultations were offered to the patients. However, there were some restrictions on the mode of delivery of the services. For example, the MUR service had to be delivered face-to-face.
**7. Locality**	The referral service was offered only to the hospitalised patients, and when a CP intervention was required, it was delivered to the patient either in the pharmacy within a private consultation room or in the patient's home.
**8. Dose and frequency**	The admitted patient was only approached once by the HPS consented to the referral to a CP. After community pharmacists had seen the referral, it was recommended they tried to contact the patient within three working days of receiving the referral. They would contact the patient, as appropriate; there was no specified number of times to contact the patients because it depended on the patient's response and the community pharmacist's practice. Furthermore, there was no time-frame to complete the referral records and no commitment to deliver the intervention and record the intervention data.<br><br>The number of CP intervention sessions and the timeframe between the sessions depended on the patients' need and type of interventions. For example, for the NMS, the patient would have the initial encounter, and then an intervention session, usually 7-14 days after the engagement. After that, the patient would have a follow-up session 14-21 days after the intervention, but this was not recorded in the PharmOutcomes platform.
**9. Tailoring or personalised**	The referral service considered the patient-centred approach where it was offered to the admitted patients according to their needs for post-discharge care. In addition, the subsequent CP services were personalised and tailored to the patients' need according to the community pharmacist's clinical judgement and upon the patient's request.
**10. Modifications or adaptation**	The referral template was modified through the course of the delivery (2016) to reduce the amount of data entered manually by the hospital staff. So more data fields were adapted so they would auto-populate with data.

Item	Description
**11. How well (planned fidelity)**	In the current referral system, only service leaders with administration rights have access to PharmOutcomes® monthly basis reports to follow-up CP response to the referral requests. The data from the platform does not allow fidelity to be well captured, but the follow-up monitoring of the service leaders checked adherence to protocol and data entry. This process ceased two years into service delivery due to the workload required.
**12. How well (actual fidelity)**	There was no record of the total number of patients that had been approached for a referral or the number of patients who actually agreed to be referred to their CP. Only one quantitative evaluation of the service was undertaken by Nazar *et al.,* which involved two hospital sites in Newcastle-upon-Tyne and 207 CPs. The evaluation assessed the referral rate by HPS, the completion rate of the community pharmacists, the type of CPs that were engaged with the service, the patients' characteristics, such as age groups and medical conditions, and the referral characteristics, such as the type of CP interventions that were requested or delivered to the patients.

Abbreviations; CP = community pharmacy, HPS = hospital pharmacy staff, LPC = Local Pharmaceutical Committee, MUR = medicine use review, NMS = new medicine service.
