# Supplementary material for: A study to investigate the implementation process and fidelity of a hospital to community pharmacy transfer of care intervention
Source: PLoS One. 2021 Dec 28;16(12):e0260951. doi: 10.1371/journal.pone.0260951 (PMC8714098; doi:10.1371/journal.pone.0260951)
Supplement: S2 Checklist — (PDF) [file pone.0260951.s002.pdf]

Additional file F: Consolidated criteria for reporting qualitative studies (COREQ).

No.	Item	Comments
**Domain 1: Research team and reflexivity**		
**Personal characteristics**		
1.	Interviewer/ facilitator	Sarah M Khayyat (SMK)
2.	Credentials	MSc
3.	Occupation	PhD student
4.	Gender	Female
5.	Experience and training	SMK attended different training sessions/courses on using interviews in qualitative research to support the effective delivery of the interviews. SMK sought support from the supervisory team, who have extensive experience of interviewing patients
**Relationship with participants**		
6.	Relationship established	SMK had no prior relationships with any of the participants
7.	Participant knowledge of the interviewer	None of the participants knew the interviewer. PTMs did know HN through her membership on the project team
8.	Interviewer characteristics	The researchers are all pharmacists with keen interests in medicines, and medicine use. This does make the research team very aware and attuned to looking for comments and phrases pertaining to these areas.
**Domain 2: Study design**		
**Theoretical framework**		
9.	Methodological orientation and theory	Thematic framework analysis that is underpinned by adapted grounded theory. Analysis proceeded via a constant comparative approach.

No.	Item	Description	Section #
**Participant selection**			
10.	Sampling	PTMs were purposively selected due to the relative low potential informants to recruit.<br>HPS, CPs and PPs were conveniently sampled through inviting participants to volunteer.	
11.	Method of approach	PTMs, HPS and CPs were all approached via email. PPs were not directly approached but the research study was advertised on national public and patient websites.	
12.	Sample size	3 PTMs, 10 HPS, 9 CPs and 11 PPs in total	
13.	Non-participation	No participants dropped out. All participants who were asked to participate agreed to do so	
**Setting**			
14.	Setting of data collection	PTMs: 2 in a meeting room in a university, 1 by telephone<br>HPS, CPs and PPs; by telephone	
15.	Presence of non-participants	No	
16.	Description of sample	This is provided in additional file F	

No.	Item	Comments	
**Data collection**			
17.	Interview guide	The authors wrote the questions and prompts were given during the interviews if needed. No pilot testing was undertaken	
18.	Repeat interviews	Yes repeat interviews (n=2) were undertaken with PTMs to verify and check data emerging from interviews with HPS and CPs	
19.	Audio/visual recording	Audio recording and verbatim transcription	
20.	Field notes	Field notes and reflection log were used after each interview	
21.	Duration	The average length of the interviews was 47 mins ±14 mins	
22.	Data saturation	Yes within the manuscript methods	
23.	Transcripts returned	No this was not carried out	
**No.**	**Item**	**Description**	**Section #**
**Domain 3: analysis and findings**			
**Data analysis**			
24.	Number of data coders	Two [HN and ZN]	
25.	Description of the coding tree	No this is not provided given the range of stakeholders interviewed. This is available on request.	
26.	Derivation of themes	A combination of thematic inductive and deductive analysis was employed as articulated in the manuscript methods	
27.	Software	Nvivo12 computer software	
28.	Participant checking	No, no member checking was undertaken	
**Reporting**			
29.	Quotations presented	Yes, this is provided within the results	
30.	Data and findings consistent	Yes	
31.	Clarity of major themes	Yes this is clearly depicted in figure 3.	
32.	Clarity of minor themes	No, this paper does not present the minutia of themes, as a system perspective is presented. This can be provided on request and is likely be reported in a publication in due course.	
