# Supplementary material for: A study to investigate the implementation process and fidelity of a hospital to community pharmacy transfer of care intervention
Source: PLoS One. 2021 Dec 28;16(12):e0260951. doi: 10.1371/journal.pone.0260951 (PMC8714098; doi:10.1371/journal.pone.0260951)
Supplement: S1 File — (DOCX) [file pone.0260951.s003.docx]

**Coding frameworks**

**Table 1. Coding framework for the interviews with the project team members.**

| **Theme** | **Categories** | **Codes** |
| --- | --- | --- |
| Service criteria  and characteristics | Number of referrals | Low referral rate, number of referrals increased after service adaptation. |
|  | Type of referrals | Change in medication, referral for specific patients, Medibox referral, MUR, NMS, other referral criteria. |
|  | Standard operating procedures (SOPs) | SOPs for community pharmacists, SOPs for HPS. |
|  | Promotions or motivations for providing the service | Yes, no. |
|  | Referral materials | Patients leaflets. |
| Beliefs of the service value | Value and benefits of the service | Convenient environment, patient support, patient safety, improve communication, reduce errors, NHS benefits, economic value. |
| Implementation process | Before implementation | Idea development, initial communication, pre-implementation meetings, other referral systems, stakeholder involved, plans for the service implementation, required permissions, resources availability (money, computer, staff, time). |
|  | During implementation | Engagement meetings, one-way communication between hospitals and CPs, tow-way communication between hospitals and CPs. |
|  | After implementation | Expectation of the implementation process, expectation of the service value, service adaptation (reason for adaptation, criteria, problems developed after the adaptation), quality of the referral information, quality of the communication, quality of the CP interventions. |
| Service barriers | Project leaders-related barriers | Hospital trusts poor engagement, lack of follow-up by the leaders or implementers, lack of follow-up by the organisations’ line managers. |
|  | HPS-related barriers | HPS understanding and appreciation of the service, lack of feedback or report on the service, staff forget to refer patients, no enough staff or no skilled staff, staff turnover, time-pressure before discharge, workload. |
|  | CP level of engagement | Poor engagement, pharmacists’ lack of appreciation/understanding of the service, staff turnover. |
|  | Patients engagement barriers | Awareness of the service, patient consent/agreement, service acceptability problems, service suitability problems. |

***Abbreviations:*** *MUR,* *Medicines Use Review;* *NMS, New Medicines Service;* *SOP, Standard operating procedure; HPS, hospital pharmacy staff; NHS, National Health Service; CP, community pharmacy.*

**Table 1. Coding framework for the interviews with the project team members (Cont.).**

| **Theme** | **Categories** | **Codes** |
| --- | --- | --- |
| Improvement strategies | Improve leaders involvement | Improve communication with community pharmacists and HPS, service monitoring, staff motivation, increase awareness of the service. |
|  | System and process improvements | Filter the type of referrals, tow-way communication, improve service reports, mandatory referrals for all patients, polices improvements, standardise the referral system across all hospitals. |
|  | Staff-related improvements | CP feedback on the referrals, increase CP engagement, involve non-pharmacy staff, provide updating and refreshing workshops. |
|  | Patients-related improvements | Increase patient awareness, service leaflet, TV service, others. |
|  | Service evaluation | Impact of CP interventions, quantitative studies, other evaluation of the service effectiveness. |

***Abbreviations:*** *HPS, hospital pharmacy staff; CP, community pharmacy.*

**Table 2. Coding framework for the interviews with hospital pharmacy staff.**

| **Theme** | **Categories** | **Codes** |
| --- | --- | --- |
| Description of the service | Referral process | Identify patients, introducing the service to the patients, patient agreement and consent, identify patient’s preferred CP, explain next action by CP, information input, materials used in the referral process. |
|  | Type of referrals | Medibox referrals, update patient’s information, request for certain services, referral for specific patients, other referral criteria, the participant is not aware of other service uses. |
|  | Awareness of SOP or service policies | Yes, no, SOP is not clear |
|  | Participant training/background to provide the service | Formal training, informal training. |
|  | Service limitation | Unidentified patients, other limitations. |
| Implementation and operation of the service | System adaptation | Less time-consuming, not time-consuming, technical changes, new system format. |
|  | Communication between HPS | Good communication, not frequent, communication does not improve the service provision. |
|  | Resources availability | Before adaptation: space and computers, staff, time, training and workshops, others.  After adaptation (new system): space and computers, staff, time, training and workshops, others. |
| Participant beliefs and awareness | Aim for referring the patients | Continuity of care and patient support, ensure patient safety, ensure patient’s understanding, improve communication and transfer of information between healthcare settings, reduce re-admissions, provide a convenient environment and 2^nd^ source of information for the patient. |
|  | Value and benefits of the referral service | Easy and quick transfer of information, ensure continuity of patient care after being discharged, good practice, improve communication and transfer of information between healthcare providers, medication adherence, fulfil patient’s need, reinforce understanding and support, patient safety and errors reduction, reduce re-admissions, source of information and documentation. |
|  | Quality of the referral process | Quality of the referral process. |
|  | Complexity of the system or referral process | Complicated, not complicated. |
|  | Evidence availability for the effectiveness of the service | Need more data or evidence, not aware of any paper or quantitative study, patient cases, there is an evidence (pharmacy reports or related papers). |
|  | Awareness of other similar services | GP letters, MAR chart, NHS.net or NHS mail, paper-based system or letters, phone calls, not aware of any similar service, others. |

***Abbreviations:*** *CP, community pharmacy; SOP, Standard operating procedure; HPS, hospital pharmacy staff; GP, general practitioner; MAR, medicine administration record; NHS, National Health Service.*

**Table 2. Coding framework for the interviews with hospital pharmacy staff (Cont.).**

| **Theme** | **Categories** | **Codes** |
| --- | --- | --- |
| Practice variation | Referral process | Different processes and information sent by HPS, variations in explaining the next expected action by CPs, variations in the way of introducing the service to the patients, time of sending the request. |
|  | Nature of the referral information (Quality) | Quality of information sent, sending open requests to CPs, sending specific requests to CPs. |
|  | Sending the discharge medication list | For all referrals, only when needed, for Medibox referrals, perceived reasons for sending incomplete medication list. |
|  | Follow up and tracking Process | Cancellation process of the referral request, HPS tracking the referral request (using the referral platform), HPS phone call tracking of the referral request. |
| CP feedback and response | CP response | CP calls, CP action, CP follows up. |
|  | Availability of reports and feedback from CP | No feedback, not aware of any CP feedback, one-way communication, occasional feedback. |
| Difficulties and problems in using the new and old referral system | Old system (before adaptation) | Data entry issues, identifying patient’s unique number, time-consuming, username and password issues, forget to provide the service, technical difficulties, system format issues. |
|  | New system (adapted system) | Data protection issue to provide additional information, identifying patient’s unique number, forget to provide the service, system format issues, no problem. |
| Service barriers | Barriers related to HPS | Resources-related barriers: additional workload, lack of training and workshops, staff availability and turnover, time pressure.  Personal barriers: appreciation of the service (not a priority), HPS Forgetting to refer patients, HPS understanding of the service uses, no motivation, the lack of service monitoring by the leaders, the delegation of tasks from one HPS to another. |
|  | Barriers related to community pharmacists | Community pharmacists’ lack of understanding and appreciation of the service, communication difficulties, referral information is not clear/incomplete, patient consent issue, permission and legislation issues, shortage of resources in CPs. |
|  | Patient’s engagement barriers | Patient acceptability of the service, patient awareness of the referral and CP services, service suitability and accessibility by all patients, communication issues, patient feedback, patient turnover. |

***Abbreviations:*** *HPS, hospital pharmacy staff; CP, community pharmacy.*

**Table 2. Coding framework for the interviews with hospital pharmacy staff (Cont.).**

| **Theme** | **Categories** | **Codes** |
| --- | --- | --- |
| Improvement strategies | Past or current strategies | Individual feedback/performance review, staff motivation, performance target, the role of media, HPS and CP feedback to adapt the system, training sessions, the participant is not aware of any strategy applied. |
|  | Service evaluation | Service evaluation. |
|  | Improvement strategies related to the patients | Increase patients’ involvement, patient education. |
|  | Improvement strategies related to the staff | Increase resources, equality in providing the service, follow up with GP pharmacists, involving or informing non-pharmacy staff about the service, financial incentive, other promotions/motivations. |
|  | System improvements | Consider some changes in the referral template, improve the discharge letter, update SOPs and other materials, other technical changes. |
|  | Obtain CP feedback | Agree, disagree, depending on the situation. |

***Abbreviations:*** *HPS, hospital pharmacy staff; CP, community pharmacy; GP, general practitioner; SOP, Standard operating procedure.*

**Table 3. Coding framework for the interviews with community pharmacists.**

| **Theme** | **Categories** | **Codes** |
| --- | --- | --- |
| Description of the service | Referral process | Request notification, CP response to the request, type of information received, communication with the GP, referral rate, referral completion rate. |
|  | Type of referrals | Medibox referral (for Information only), MUR, NMS, referral from and to the GP, other support. |
|  | Awareness of SOP or service policies | Yes, not aware of the SOP, not aware of some procedures, importance of using the SOPs. |
|  | Participant experience and training background to provide the service | Experience from one site, experience from different sites, experience in years. |
|  | Care for patients with T2DM | Patients are followed by other healthcare professionals, no different care, special care for patients on insulin. |
| Implementation and operation of the service | System adaptation | Aware of the system adaptation, not aware of any adaptations. |
|  | Communication with others | Between community pharmacists each other, between hospitals and CPs, communication with project leaders. |
|  | Resources availability | Computers, consultation room, staff, time, training, money. |
| Participant beliefs and awareness | Aim for referring the patients | Improve communication and transfer of information between healthcare settings, part of the business or job, patient support. |
|  | Value and benefits of the referral service | Better workflow, improve communication and transfer of information between healthcare providers, ensure continuity of patient care after being discharged, patient support, patient safety and errors reduction, reduce hospital re-admissions, provide convenient environment and source of information/documentation. |
|  | Quality of the referral process | Can be improved, others. |
|  | Complexity of the system or referral process | Not complex. |
|  | Evidence availability for the effectiveness of the service | For patients with T2DM, no evidence. |
|  | Awareness of other similar services | Faxes, letters by post, NHS 111, NHS mail or chat box, phone calls. |

***Abbreviations:*** *CP, community pharmacy; GP, general practitioner; MUR, Medicines Use Review; NMS, New Medicines Service; SOP, Standard operating procedure; T2DM, type 2 diabetes mellitus; NHS, National Health Service.*

**Table 3. Coding framework for the interviews with community pharmacists (Cont.).**

| **Theme** | **Categories** | **Codes** |
| --- | --- | --- |
| Practice variation | Nature of the referral information (Quality) | Quality of information sent, sending open requests to CPs, sending specific requests to CPs, barriers for the specific requests, reasons for sending incomplete information to CPs. |
|  | Follow up Process | Discussing medication changes with the patient: yes, no. |
| CP feedback and response | CP actions or follow up | Interventions delivered to the patients directly: post-discharge phone call, MUR, home MUR, NMS, initiate Medibox or other aids.  Follow up interventions: delivery service, medication double checking, providing medication leaflets and other supporting materials.  No action or service provided, other services provided in the CP. |
|  | Guideline to provide CP services | Available, not available. |
|  | Availability of reports and feedback from CP | CP sent feedback to hospitals, no feedback, one-way communication, reasons for not sending feedback to the hospitals. |
|  | CP data entry | Recorded consultation and services, not recorded (Medibox referral), not recorded (data recorded in the PMR), other reasons for not completing the referral template, other free-type information. |
| Difficulties and problems in using the referral system | Communication-related issues | Non-standardised system across all hospitals, service is underused, CPs did not receive PharmOutcome notification. |
|  | Data-related issues | Difficulty in identifying patient’s ward, inaccurate information, information does not match the GP’s letter, missing or not clear information, data protection issue to provide additional information, additional information is needed. |
|  | System format issues | Font size, layout issue. |
| Service barriers | Barriers related to HPS | HPS understanding of the service uses. |
|  | Barriers related to community pharmacists | Community pharmacists understanding of the service uses, permissions and legislation, mode of delivery (face-to-face or phone call), patient consent issues. |
|  | CP resources-related barriers | Additional workload, computers, lack of training, money, staff availability and turnover. |
|  | Patient’s engagement barriers | Patient acceptability, patient awareness of the referral and CP services, accessibility issue, non-contactable patients, housebound patients, service suitability, communication issues. |

***Abbreviations:*** *CP, community pharmacy; MUR, Medicines Use Review; NMS, New Medicines Service; PMR, patient medical record; GP, general practitioner; HPS, hospital pharmacy staff.*

**Table 3. Coding framework for the interviews with community pharmacists (Cont.).**

| **Theme** | **Categories** | **Codes** |
| --- | --- | --- |
| Improvement strategies | Past or current strategies | Filtering question about the type of referrals, not aware of any improvement strategy. |
|  | Service evaluation | Evaluators need CP feedback, referral service evaluation, CP interventions evaluation, others. |
|  | Improvement strategies related to the patients | Obtain pre-discharge patient consent, provide phone MUR, increase patient involvement, patient education, improve referral criteria. |
|  | Improvement strategies related to the staff | Staff training, involve the diabetic clinics’ staff in the referral process, involve GPs in the referral process. |
|  | System improvements | Attach discharge letter, improve referral template layout. |
|  | External influence to provide the service | Discuss benefits of the service, provide feedback or service evaluation, improve service providers and commissioners involvement. |

***Abbreviations:*** *CP, community pharmacy; MUR,* *Medicines Use Review, GP, general practitioner.*

**Table 4. Coding framework for the interviews with patients and members of the public.**

| **Theme** | **Categories** | **Codes** |
| --- | --- | --- |
| Awareness of CP  services and referral service | Awareness of CP services | Dispensing role, prescribing role, counselling services, medication review, other services, not aware of the available CP services, what does community pharmacy mean?, available posters and adverts in the pharmacy. |
|  | Awareness of the referral service | Aware of the service: from the GP, community pharmacists explained the service, national press, previous experience for a family member, reading about the service.  Not aware of the service, little information is known about the service. |
| Appreciation of CP services and referral service | Appreciation of CP services | Yes , no, others. |
|  | Appreciation of the referral service | Patient’s acceptance of the service, patient’s willingness to be referred, others. |
| Benefits of the CP services and the referral service | Benefits to the community pharmacists | Pharmacists will have the potential to provide more specific advice on medication, provide more services, easy to contact and communicate with the patient. |
|  | Benefits to the NHS | Easy transfer of information, improve communication between healthcare professionals after discharge, reduce pressure on GP and hospital services, reduce re-admissions, economic value. |
|  | Benefits to the patients | Continuity of care, patient safety, improve medication adherence, provide reassurance to the patients, direct patient referrals to other healthcare professionals, the impact of the service on long-term conditions, the impact of the service on short-term conditions |
| Difficulties and barriers associated with using the referral and CP services | Problems related to CPs | Accessibility problems related to the pharmacy premises, different CP provide different services, no private consultation room, bad opening hours, prescription preparation takes time, no problem reported by the participant. |
|  | Problems related to the referral system or healthcare system | Different health systems exist, security and confidentiality issues, patient consent issues, other sharing information issues, issues related to using the electronic referral system. |
|  | Problems related to the patients | Communication difficulties between patient and community pharmacist, lack of appreciation of CP services, lack of awareness and understanding of CP services, patients acceptance/rejection of the referral service, reluctant to change (GP should be first port of call). |
| Stratgies to increase  awareness of the referral and CP services | Staff involvement | Involve non-HPS and hospital consultants, involve GPs, engage other staff (like receptionist). |
|  | Increase awareness | Word of mouth, publicise the word community pharmacy, provide evidence of the service benefits, campaign, digital screen/TV, emails, patient groups, leaflets, posters, discharge package, online information, NHS website, message reminders, social media, news, national and local press. |

***Abbreviations:*** *CP, community pharmacy; GP, general practitioner; NHS, National Health Service;* *HPS, hospital pharmacy staff, NHS; National Health Service.*
