# Supplementary material for: A study to investigate the implementation process and fidelity of a hospital to community pharmacy transfer of care intervention
Source: PLoS One. 2021 Dec 28;16(12):e0260951. doi: 10.1371/journal.pone.0260951 (PMC8714098; doi:10.1371/journal.pone.0260951)
Supplement: S1 Guide — (PDF) [file pone.0260951.s004.pdf]

Additional File A. The interview guide with the project team members

**Opening the interview**	
- Greet the participants and thank them for taking part in the research.<br>- Explain again the purpose of the study.<br>- Ask participants if they would like to ask any question before starting the interview.<br>- Emphasise to the participants that there is no right or wrong answer and we are just interested in their experiences.<br>- Discuss the participant information sheet, if the participant has not read it in advance.<br>- Discuss the participant informed consent and ensure it is signed.<br>- Complete the demographic form.<br>- Check the audio recorder and ask the participants if they are happy to begin the interview.<br><br>Before we start this interview, I would like to confirm you know that:<br>1. Your participation in this study is completely voluntary.<br>2. You are free to refuse to answer any questions.<br>3. You are free to withdraw but only up to the conclusion of the interview.<br>4. The interview will be strictly confidential and anonymised and all information disclosed during this interview will only be available to the research team. Excerpts from this interview may be part of the final report of the project. However, information used in the project report will NOT be linked back to you. All reports and information collected will be stored securely at Newcastle University.<br><br>Are you ready to proceed with the interview?	
**Body of the interview and research questions**	
The interview questions would explore the electronic transfer of care (eToC) service. It will cover the implementation process; before, during and after initiation of the service. The project leaders' perception about the use of the service and its quality would be covered as well.	
**a. Before service implementation (plan)**	1. Can you tell me how you first came to be involved with the eToC service?<br>**Prompt:** When was this? And what happened next?<br><br>2. What were the reasons for developing this service?<br><br>3. How did you develop the idea? Plan for the service implementation? (e.g., to start applying it in the North-East, certain hospitals or wards)?

**b. During the implementation**	1. What did you do to engage staff from hospitals in the process of implementation?<br>**<u>Prompt:</u>**<br>- What about the staff from community pharmacies?<br>- How were they informed about the implementation of the new service?<br>- Have you distributed a softcopy or hardcopy of the service policies/standards to the engaged hospital pharmacy staff and community pharmacists?<br>- What was their level of engagement?<br>(i.e., indicators for their commitment to implementing the eToC service).<br>- What other strategies could you use to spread provision of the service?<br><br>2. Who else was involved in the implementation process?<br>(i.e., other than hospital pharmacy staff and community pharmacists)<br><br>3. How was the system when people started using it?<br>**<u>Prompt:</u>** Have there been any issues? (e.g., technical problems).<br><br>4. What was the communication like between you (as a project leader) and engaged personnel (hospital pharmacy staff/community pharmacist)?<br><br>5. What was the communication like between hospital pharmacy staff and community pharmacists?<br>**<u>Prompt:</u>** How do you think this communication would effect the quality of implementation and providing the service?<br><br>6. What do you think about the availability of the resources for implementing and providing the eToC service?<br>**<u>Prompt:</u>** What could be better?<br>(e.g., having enough money, physical space, time, and engagement of appropriate personnel in the marketing, education and training sessions).
**c. After implementation and adaptability stage**	1. How did the initial implementation go?<br>**<u>Prompt:</u>**<br>- Is it different from what you were expecting/planned for?<br>- Do you think it's working?<br>- Is there any evidence for its effectiveness?<br>(i.e., considering any similar services across the UK, studies, published papers, or other evidence).<br><br>2. What happened after that? Has the service been adapted?<br>**<u>Prompt:</u>**

	- What were the criteria for adaptation?<br>- Who decided the changes needed?<br><br>3. Have there been any other problems/concerns with the service since its adaptation?<br><br>4. Does anyone get paid for doing it? If not, would that make any difference?<br><br>5. Are there any motivations for providing the service?<br>**<u>Prompt:</u>** Would that make any difference?<br>(e.g., motivations like annual performance review, promotion and increased stature or respect).
**d. Project leaders' perception about the use of the service**	1. What is your overall feeling about this service? Its value and importance?<br>**<u>Prompt:</u>**<br>- What is the impact of the service on practice?<br>- What is the impact of the service on achieving the needs and desired outcomes in patients? (e.g., improving medication adherence/counselling, reduce hospital re-admission)<br>- What would be the barriers to meet the patient's needs?<br><br>2. What is the overall feeling about this service amongst community pharmacists and hospital pharmacy staff in the North-East?<br><br>3. What is your perception of the quality of the referring process? What would make a good ToC service?<br><br>4. Is evaluation of the service important? Why?<br>**<u>Prompt:</u>**<br>- How would you evaluate it?<br>- Are there any quantitative reports or qualitative feedback for the progression of the service?<br>- How would the community pharmacy feedback to the hospitals help in improving the service?<br>- Have you got any feedback?<br><br>6. What would you do if you could improve it? Are you planning to adapt the service again?
**Closing the interview**	
- Ask the participants if they would like to add or ask about anything else before closing the audio recorder and finishing the interview.<br><br>- Thank the participant again for taking part in the study.	
