# Supplementary material for: A study to investigate the implementation process and fidelity of a hospital to community pharmacy transfer of care intervention
Source: PLoS One. 2021 Dec 28;16(12):e0260951. doi: 10.1371/journal.pone.0260951 (PMC8714098; doi:10.1371/journal.pone.0260951)
Supplement: S2 Guide — (PDF) [file pone.0260951.s005.pdf]

Additional file C. The interview guide with hospital pharmacy staff

<table>
<tr><th colspan="2">Opening the interview</th></tr>
<tr><td colspan="2">
- Greet the participants and thank them for taking part in the research.
- Explain again the purpose of my study.
- Ask participants if they would like to ask any question before starting the interview.
- Emphasise to the participants that there is no right or wrong answer and that I am just interested in their experiences.
- Discuss the participant information sheet, if the participant has not read it in advance.
- Discuss the participant informed consent and ensure it is signed.
- Complete the hospital pharmacy staff demographic form.
- Check the audio recorder and ask the participants if they are happy to begin the interview.

Before we start this interview, I would like to confirm you know that:
- Your participation in this study is completely voluntary.
- You are free to refuse to answer any questions.
- You are free to withdraw but only up to the conclusion of the interview.
- The interview will be strictly confidential and anonymised and all information disclosed during this interview will only be available to the research team. Excerpts from this interview may be part of the final report of the project. However, information used in the project report will NOT be linked back to you. All reports and information collected will be stored securely at Newcastle University.

Are you ready to proceed with the interview?
</td></tr>
<tr><th colspan="2">Body of the interview and research questions</th></tr>
<tr><td colspan="2">
The questions would be about the electronic transfer of care (eToC) service. The interview will consist of two parts which are:
a. The implementation of the service and its barriers and facilitators
b. The characteristics of the eToC service.
</td></tr>
<tr><td>a. The implementation stage and barriers and facilitators of the electronic referral process at the hospital</td><td>
➢ The intervention characteristics
1. What are the advantages of implementing the eToC service?

2. When the service was first started, what were the main difficulties in providing it? What are the current difficulties?
(e.g., having any communication difficulties with community pharmacies? Time-consuming? Workload?).

3. How complicated is this process?

4. What is your perception about the quality of the referring process? What would make a good eToC service?
</td></tr>
</table>

	➢ **The outer setting**<br>1. How do you think this service meets patients' needs?<br><br>2. What would be the barrier and facilitators of meeting the patients' needs?<br><br>3. What strategies does the hospital use to spread the use of the eToC service (e.g., application of policies and regulations)?<br><br>4. What other external strategies applied/do you suggest to spread the use of the service?
	➢ **The inner setting**<br>1. How do good/poor networks and communications between hospital staff affect the implementation/provision of the eToC service?<br><br>2. How would the motivations and payment for providing the service effect the use of the service and referring patients?<br>(e.g., extrinsic motivations like annual performance review, promotion, raises in salary and increased stature or respect).
	➢ **The characteristics of the involved individuals**<br>1. What are your beliefs about the value and importance of the eToC service?<br><br>2. Are there any similar services across the UK, studies or published papers that prove the effectiveness of such a service?
	➢ **The process of implementation**<br>1. What do you think about the availability of the resources for implementing and providing the eToC service?<br>(e.g., having enough money, physical space, time, and engagement of appropriate personnel in the marketing, education and training sessions).<br><br>2. What is the importance of evaluating the progress and quality of the service?<br><br>3. How would you evaluate the service? (e.g., quantitative, qualitative feedback and performance reviews).

**b. The characteristics of the referral process**	1. What selection criteria do you use to refer patients?<br><br>2. What are the reasons for referring those patients?<br>What would you prefer, sending a general request to community pharmacy (e.g., review with the patient), or specific request on a particular issue?<br><br>3. What is your rationale/goal of providing the electronic referral service? (e.g., to improve the continuity of care).<br><br>4. What materials do you use to refer the patients? (e.g., leaflets/documents, and a brief chat with the patients).<br><br>5. Can you please explain to me how do you usually refer patients using PharmOutcomes?<br>OR describe the mode of delivery of the service (e.g., identifying the patient's eligibility criteria, and obtaining patients' informed consent … etc.).<br><br>6. Can you tell more about your background/training you had to use the referral system/PharmOutcomes?
**Closing the interview**	
- Ask the participants if they would like to add or ask about anything else before closing the audio recorder and finishing the interview.<br><br>- Thank the participant again for taking part in the study.	
