# Supplementary material for: A study to investigate the implementation process and fidelity of a hospital to community pharmacy transfer of care intervention
Source: PLoS One. 2021 Dec 28;16(12):e0260951. doi: 10.1371/journal.pone.0260951 (PMC8714098; doi:10.1371/journal.pone.0260951)
Supplement: S3 Guide — (PDF) [file pone.0260951.s006.pdf]

Additional file D. The interview guide with community pharmacists

Opening the interview

- Greet the participants and thank them for taking part in the research.
- Explain again the purpose of my study.
- Ask participants if they would like to ask any question before starting the interview.
- Emphasise to the participants that there is no right or wrong answer and that I am just interested in their experiences.
- Discuss the participant information sheet, if the participant has not read it in advance.
- Discuss the participant informed consent and ensure it is signed.
- Check the audio recorder and ask the participants if they are happy to begin the interview.

Before we start this interview, I would like to confirm you know that:

- Your participation in this study is completely voluntary.
- You are free to refuse to answer any questions.
- You are free to withdraw but only up to the conclusion of the interview.
- The interview will be strictly confidential and anonymised and all information disclosed during this interview will only be available to the research team. Excerpts from this interview may be part of the final report of the project. However, the information used in the project report will NOT be linked back to you. All reports and information collected will be stored securely at Newcastle University.

Are you ready to proceed with the interview?

Body of the interview and research questions

The questions will about the electronic transfer of care (eToC) service and any subsequent interventions provided to patients here in community pharmacy.
The participant brief introduced what we mean by 'eToC' and 'intervention' in this study context, are you clear about those descriptions as I will use those terms throughout? I may make particular reference to patients with type 2 diabetes mellitus (T2DM) at times.
The interview will consist of four parts which are:

a. The implementation/operation of the service and any barriers and facilitators to the service, and/or to delivering community pharmacy interventions
b. The characteristics of the community pharmacy interventions themselves
c. Effectiveness and quality of the service
d. Service evaluation and potential improvement.

**a. The implementation/ operation of the service and any barriers and facilitators to the service, and/or to delivering community**	TO START:<br>1. Can you tell me when you first started delivering this eToC service?<br>**Prompt:**<br>- Did you start when the service started 4 years ago or have you taken it on since?<br>- Have you always delivered from this pharmacy or elsewhere?<br><br>2. How were you recruited to deliver it?

pharmacy interventions	3. Do you have now, or have you ever had, a copy of the service specification/ policies/ standards (electronic or hard copy)?<br><br>4. What training have you had to deliver the eToC service (formal or on the job training)?<br><br>5. What is your experience of implementing/delivering this service?<br>**Prompt:**<br>- Are you able to describe any positive experiences?<br>- Did you have any issues with providing the service? (e.g., any technical problems, difficulty in using the system, time constraint, workload).<br><br>6. Do you know of any adaptations made to the service since it first started about four years ago?<br>**Prompt:** If yes, were the changes difficult to make? Have these helped/hindered service provision?<br><br>7. What first provided the motivation for you to deliver this service?<br>**Prompt:** Is that motivation the same now?<br><br>8. What do you think about resourcing of the service and any associated interventions?<br>**Prompt:** Only if do not answer (e.g., having enough money, physical space, time, and engagement of people in the marketing/promotion of it, education and training sessions).<br><br>9. Of the interventions you do or could provide to patients following an eToC referral, do you receive a payment for any of these (directly or indirectly)?<br>**Prompt:**<br>- We understand you can be paid if you deliver an MUR or NMS, but do you get paid for providing other interventions/services to the referred patients?<br>- Do you think you could be paid for any part of the intervention response? (e.g., for filling in a record of your intervention to give feedback to the hospital pharmacy?)<br>- How would this affect either your motivation or ability to complete an intervention and/or to record that you have done that intervention?
**b. The characteristics of the community pharmacy**	1. How often do you receive referrals from the eToC system on PharmOutcomes?<br>**Prompt:** A referral each day, each week, each month etc.

**interventions themselves**	2. And what proportion of these would you follow up with an intervention? (this confirms frequency of intervention in this pharmacy)<br><br>3. When you do not follow up on any individual referral with an intervention, what would be the reasons for this?<br><br>4. If you follow up the referrals with an intervention what would make you follow them up? (reason or goal, e.g., poor adherence, continuity of care)<br><br>5. Would you always record this in PharmOutcomes?<br>**<u>Prompt:</u>**<br>- Do you populate/document the data in PharmOutcomes every time you provide an intervention?<br>- If not, why not?<br><br>6. When you receive a Medibox or "For Your Information (FYI)" referral, how do you deal with it?<br>**<u>Prompt:</u>**<br>- What does FYI mean to you?<br>- Do you discuss the changes with the patient?<br><br>7. What type of interventions do you make?<br>**<u>Prompt:</u>** To clarify, interventions could include any active response that you make to the PharmOutcomes® referral request such as: an MUR, NMS, a phone call to GP, follow up phone call with the patient, patient counselling/education or making a note on the patient medical record.<br><br>8. Where and how do these take place?<br>**<u>Prompt:</u>** Do these take place in a face-to-face visit or by a phone call? In the pharmacy or the patient's home?<br><br>9. Do you provide any informational materials to the patient?<br>**<u>Prompt:</u>**<br>- Do you use any material/resources to deliver the service/intervention, like the use of guidelines?<br>- Where the materials can be accessed? (e.g., online appendix, websites).
**b. Effectiveness and quality of the service**	1. What is your overall opinion of the eToC service, in principle? Is it valuable, if so, to whom?

	2. Do you think that the existence of the eToC service has had any impact on your practice with patients?<br>**Prompt:**<br>- If yes, how? Does it improve the adherence/counselling, reduce hospital readmission, save money and improve patient safety?<br>- Does this service make any difference compared to the discharge process before implementing the eToC, or to patients who were not referred via eToC?<br>(e.g., reducing transcription errors/communication error when telling medications changes over the phone)<br>- If not, why not, what would need to be different for it to have a positive impact on patients? (barriers to it working/facilitators)<br><br>3. Is there any particular or different impact for patients with different conditions?<br>Thinking about the nature of interventions provided/could be provided to patients?<br>**Prompt:** How could community pharmacist intervention following an eToC referral have a real impact to improve patients' health and potentially reduce their likelihood of hospital re-admission?<br><br>4. What is your perception of the quality of the referral from the hospital side?<br>**Prompt:**<br>a. What would make a good eToC service?<br>b. Do you get the kind of information you need from hospital pharmacy staff to follow-up the patients?<br>c. Is there any information missing, if so what is the most important missing information you need? Do you need the full discharge medications list for all referred patients? Or only when required?<br>d. How could any missing information (e.g., incomplete medication list) affect the quality of the service and patient outcomes (e.g., reducing hospital re-admission)?<br>e. What do you think might be the reasons for incomplete referral information coming from hospital pharmacy staff? (e.g., incomplete list of medications)<br><br>5. Does communication between hospital pharmacy staff and community pharmacists affect the quality of service delivery/referral? Or delivering a suitable intervention?

	6. What would you prefer, being directed by the hospital staff about what intervention/care the patient needs or using your own professional judgement with each patient? Why is that?<br><br>7. Have you sent any feedback to the referring hospitals? How?<br>**<u>Prompt:</u>**<br>- If not, what are your reasons?<br>- Could having community pharmacy feedback improve the service? (i.e., by means of an on-off evaluation OR with a quick tick-box feedback record of what community pharmacy did after each referral?)
**c. Service evaluation and potential improvement**	1. Would you recommend an evaluation of the service? Why?<br>**<u>Prompt:</u>**<br>- Are there any quantitative reports or qualitative feedback for the progression of the service OR any evaluation reports that have been completed/shared with you? OR hearsay amongst your colleagues?<br><br>2. How or what would you like to see evaluated about the service and/ or interventions provided?<br>3. Do you think it would be valuable to increase the use of/spread the uptake of this service?<br>**<u>Prompt:</u>** If yes, what key message or external influences could be used to extend it/promote it?<br><br>GO TO CLOSE THE INTERVIEW
**Closing the interview**	
- Ask the participants if they would like to add or ask about anything else before closing the audio recorder and finishing the interview.<br><br>- Thank the participant again for taking part in the study.	
