# Supplementary material for: A study to investigate the implementation process and fidelity of a hospital to community pharmacy transfer of care intervention
Source: PLoS One. 2021 Dec 28;16(12):e0260951. doi: 10.1371/journal.pone.0260951 (PMC8714098; doi:10.1371/journal.pone.0260951)
Supplement: S4 Guide — (PDF) [file pone.0260951.s007.pdf]

Additional file E. The interview guide with patient and the public

<table>
<tr><th colspan="2">Opening the discussion</th></tr>
<tr><td colspan="2">
- Greet the participants and thank them for taking part in the research.
- Explain again the purpose of my study.
- Ask participants if they would like to ask any question before starting the discussion.
- Emphasise participants that there is no right or wrong answer and that I am just interested in their experiences.
- Discuss the participant information sheet, if the participant has not read it in advance.
- Discuss the participant informed consent and ensure it is signed.
- Complete the patient’s demographic form.
- Check the audio recorder and ask the participants if they are happy to begin the discussion.

Before we start this group discussion, I would like to confirm you know that:
- Your participation in this study is completely voluntary.
- You are free to withdraw but only up to the conclusion of the discussion.
- The discussion will be strictly confidential and anonymised and all information disclosed during this discussion will only be available to the research team. Excerpts from this discussion may be part of the final report of the project. However, information used in the project report will NOT be linked back to you. All reports and information collected will be stored securely at Newcastle University.

Are you ready to proceed with the discussion?
</td></tr>
<tr><th colspan="2">Body of the discussion and research questions</th></tr>
<tr><td colspan="2">
The questions will be about the transfer of care service, and the discussion will consist of four parts:
a. Patients’ awareness of the electronic referral service.
b. Patients’ acceptance/willingness to be referred to and interact with their community pharmacist post hospital discharge.
c. Patients’ positive and negative experiences/views and expectations of using the eToC service and post-discharge community pharmacy services.
d. The difficulties and challenges associated with being unable to visit the CP and use the service.
</td></tr>
<tr><td>a. Patients’ awareness of the electronic referral service</td><td>
1. What do you know about the transfer of care service where hospital inpatients are referred to their community pharmacy for care after they are discharged?
<u>Prompt:</u>
How much do you know about it? Can you tell me more?
2. How did you introduce/heard about it?
<u>Prompt:</u>
How do you think inpatients should be told about it?
</td></tr>
</table>

**b. Patients' acceptance/ willingness to be referred to and interact with their community pharmacist post hospital discharge**	1. What do you think about such a service being offered?<br><br>2. What are your expectations about how the service can help you or patients?<br><br>3. Do you think community pharmacists have enough knowledge and are helpful to manage patients and their condition?<br>**Prompt:**<br>How did you develop this knowledge/perception?<br><br>4. How do you perceive the care you receive in community pharmacy as opposed to the care you receive in the hospital?<br>**Prompt:**<br>Think about the flexibility of making an appointment, appropriate call timing/duration, and problem-solving.<br><br>5. What are your thoughts on this service being provided to patients with short-lasting conditions? And what about if they had a long-term condition, like asthma?....and what about diabetes?
**c. Patients' positive and negative experiences/ views and expectations of using the eToC service and post-discharge community pharmacy services.**	1. Overall, how would you describe your experience of using the electronic referral? (Think about the post-discharge continuity of care and personalised care).<br>**Positive experiences/views**<br>2. What are some of the possible benefits of the service?<br><br>**Negative experiences/views**<br>3. Have you had any negative feedback with using the service? Or do you have any issue with using it?<br>**Prompt:**<br>- Do you have any problem in sharing your hospital admission data with your registered community pharmacy? (data sharing concern)<br>- What personal information would you like/dislike to share?
**d. The difficulties and challenges associated with being unable to visit the CP and use the service**	1. What do you think might be the main difficulties in using the service?<br>**Prompt:**<br>Do you have any communication difficulties with community pharmacies?<br><br>2. What would stop you or patients from wanting to try or use one of the community pharmacy services?<br>**Prompt:**<br>- Is your pharmacy accessible to patients with disabilities? Do you manage to see your pharmacists when needed?<br>- What do you think the other reasons for not using the service?<br><br>3. What are your recommendations for future service delivery?
**Closing the discussion**	

- Ask the participants if they would like to add or ask about anything else before closing the audio recorder and finishing the interview. Thank the participant again for taking part in the study.
