# Supplementary material for: A study to investigate the implementation process and fidelity of a hospital to community pharmacy transfer of care intervention
Source: PLoS One. 2021 Dec 28;16(12):e0260951. doi: 10.1371/journal.pone.0260951 (PMC8714098; doi:10.1371/journal.pone.0260951)
Supplement: S1 Table — Demographic tables for PTM, HPS and CPs (a) and for PPs (b). (PDF) [file pone.0260951.s008.pdf]

Additional File G. Demographic tables for PTM, HPS and CPs (a) and for PPs (b)

(a)

Variables		Project team members (n=3)	Hospital pharmacy staff (n=10)	Community pharmacists (n=9)
**Age group**	Mean (SD)	44 (± 6)	34 (±11)	39 (±10)
	18 - 24 years	0	2	0
	25 - 34 years	0	4	3
	35 - 44 years	1	2	4
	45 - 54 years	2	1	1
	55 to > 65 years	0	1	1
**Employment status**	Full-time employed	3	9	9
	Part-time employed	0	1	0
	Locum pharmacist	0	0	0
**Occupation**	Pharmacists	3 (1 hospital, 1 community, 1 commissioner)	7	9
	Pharmacy technicians	0	3	0
**Work experience**	≤ 1 year	0	2	0
	2 - 5 years	0	2	3
	6 -9 years	0	1	1
	≥ 10 years	3	5	5
**Education level**	Diploma	0	1	0
	Bachelor's degree	1	3	7
	Master's degree	1	1	0
	Doctor of Pharmacy	0	0	1
	Postgraduate diploma	1	5	1
**Type of community pharmacy**	Supermarket pharmacy			0
	Independent/owned pharmacy (≤5 outlets)			2
	Small regional pharmacy (≤20 outlets)			2
	National large chain pharmacy (>20 outlets but <200)			0
	Multiple pharmacies (≥200 outlets)			5

(b)

Variables	Groups	Number of participants (n=11)
**Type of participant**	Healthy individual	4
	Patient with LTCs (hypertension, asthma, osteoarthritis, epilepsy)	4
	Carer of patients with LTCs	1
	Patient with other medical condition (non-LTC)	2
**Participant had or offered a CP service after any hospital discharge**	Yes	0
	No	8
	Do not know/cannot remember	3
**Age group**	Mean (SD)	59 (±18.6)
	18 - 24 years	1
	25 - 34 years	1
	35 - 44 years	0
	45 - 54 years	1
	55 - 65 years	3
	> 65 years	5
**Gender**	Male	4
	Female	7
**Ethnic group**	White British	10
	White European	1
**Marital status**	Single	6
	Married	3
	Divorced/Separated	1
	Widowed	1
**Employment status**	Public/government job	2
	Self-employed	1
	Retired	8
**Highest education level**	General Certificate of Secondary Education/O-levels	1
	A-level/National Vocational Qualification	3
	Diploma	1
	Degree	3
	Postgraduate	3
